# Molecular docking analysis of metformin analogues with GSK-3β

**DOI:** 10.6026/97320630018269

**Published:** 2022-03-31

**Authors:** Ponnu lakshmi Rajagopal, Vidhya Rekha Umapathy, Sandhya P, Fathima JH, Ramajayam Govindan, Chella Perumal Palanisamy, Vishnu Priya Veeraraghavan, Selvaraj Jayaraman

**Affiliations:** 1 Central Research Laboratory, Meenakshi Academy of Higher Education and Research (Deemed to be University), Chennai, India; 2 Department of Public Health Dentistry, Sree Balaji Dental College and Hospital, Pallikaranai, Chennai-600 100, India; 3 Department of Biochemistry, Allied Health science, Dr. M.G.R Educational and Research Institute, Chennai India; 4 Department of Oral and Maxillofacial Pathology, Ragas Dental College and Hospitals, Chennai, India; 5 Multi Disciplinary Research Unit, Madurai Medical College, TamilNadu, India; 6 State Key Laboratory of Bio-based Materials and Green Papermaking, College of Food Science and Engineering, Qilu University of Technology, Shandong Academy of Science, Jinan 250353, China; 7 Department of Biochemistry, Saveetha Dental College and Hospitals, Saveetha Institute of Medical and Technical Sciences, Saveetha University, Chennai, India

**Keywords:** Diabetes mellitus, GSK-3β, Metformin analogues, Molecular docking studies

## Abstract

We report the molecular docking analysis of four analogues of metformin [1-Carbamimidoyl-1,2-dimethylguanidine hydrochloride, Metformin hydrochloride, N1,N1-Dimethyl-N5-methylbiguanide hydrochloride, and N1,N1,N5,N5-Tetrakis (methyl-biguanide hydrochloride] with GSK3.

## Background:

National Health and Morbidity Surveys (NHMS) have recorded an increasing trend in DM prevalence over the last few decades [1]. The current treatment of diabetes, which includes insulin therapy and a variety of allopathic medications, has been confirmed to have significant side effects and minimal effectiveness [2]. Glycogen synthase kinase 3 is a member of the mitogen-activated protein kinase superfamily. GSK 3 has been linked to the production of insulin resistance and glycogen synthesis control [3]. It is a key target in the treatment of type 2 diabetes. Inhibitors of GSK-3 have been shown to increase insulin sensitivity, glycogen synthesis, and glucose metabolism in diabetic patients' skeletal muscles [4,5]. Metformin is a compound that belongs to the class of drugs known as biguanidines. It is the first therapeutic choice for Type 2 Diabetes Mellitus, since it inhibits hepatic gluconeogenesis and prevents hyperglycemia without impairing insulin secretion, hypoglycemia, or weight gain [6]. It has been used for more than four decades [7]. Therefore, it is of interest to report the molecular docking analysis of four analogues of metformin [1-Carbamimidoyl-1, 2-dimethylguanidine hydrochloride, Metformin hydrochloride, N1,N1-Dimethyl-N5-methylbiguanide hydrochloride, and N1,N1,N5,N5-Tetrakis (methyl-biguanide hydrochloride] with GSK3.

## Materials and Methods:

### Preparation of ligands:

The Metformin and its analogues were downloaded from PubChem database. Ten analogues structure (Table 1 - see PDF) were downloaded by using similar structure option. All the compounds were downloaded in SDF file format. And then it was converted into PDB format using online smiles translator. Finally all the analogues were converted into PDBQT file format using Auto Dock Tool (ADT) for further analysis.

### Preparation of Protein:

Three-dimensional coordinates GSK 3β (PDB: 1Q4L) were retrieved from Brookhaven Protein Data Bank. The PDB files were produced by modeling in missing side chains, performing a small amount of regularization, correcting water positions and symmetry, and adding hydrogen. Only chain A of the repaired pdb file was analyzed and the resulting pdbqt file was transferred to AutodockTools (ADT ver.1.5.6) for preparation. Thus, water molecules and non-standard residues were eliminated, leaving only polar hydrogen, and Gasteiger charges for protein atoms were computed using ADT.

### Molecular docking:

Docking protocol validation in molecular docking is necessary to ensure that ligands connect within the binding pocket in the proper conformation, which is accomplished by validating the size and centre of the coordinates of the grid box around the binding pocket [8]. PyRx was used as a simulated screening method for computational drug exploration [9] to screen the ligand files against the protein. AutoDock 4 and AutoDockVina are used by PyRx as docking methods for the Lamarckian Genetic Algorithm and Analytical Free Energy Scoring Feature. Using our choice of inhibitors, PyRx was carried out on the predicted energy-minimized protein structure. Using the PyRx platform, the macromolecular protein structure was prepared and then docked into the binding site residues within a grid box with X, Y and Z axes and measurements. The docking procedure was then worked at the exhaustiveness of 8, and set to generate only the lowest energy pose. We analyzed the relationships between our targeted protein and the ligands used to process and prepare the figures using Pymol Molecular Visualization Tools [10].

## Results and Discussion:

Molecular docking is a simulation technique that determines the optimal position for a ligand to bind to a target's active site. This technique entails selecting the three-dimensional coordinate space of the binding site in the target and measuring the binding interactions of the molecule's resultant orientation within the binding site, forming the complex. The importance and sensitivity of binding affinity values are calculated by the maximum magnitude negative number (highest binding affinity or lowest binding energy), which represents the most advantageous conformation of the complex produced when the ligand involved binds efficiently to the target's active pockets. Additionally, docking simulations were used to confirm the anti-diabetic potency of metformin analogues by examining the binding affinity and orientation of ligands inside the GSK 3 receptor pocket. The docking poses were rated as per their score values, and Table 2(see PDF) summarized the binding affinities of the best pose for each of the four analogues with the GSK 3 target (Figure 1 - see PDF) the binding affinity of complexes was determined to be between - 6.1 and 7.3kcal/mol, confirming their excellent potency. Additionally, the molecules in this analysis have a higher binding affinity, comparable to that of a regular drug (metformin).The four compounds could dock into the active site of GSK 3β successfully. The binding energies of -7.3, -6.8 and -6.6 and 6.1 kcal/mol were obtained for1-Carbamimidoyl-1,2-dimethylguanidine;hydrochloride, Metformin hydrochloride, N1,N1-Dimethyl-N5-methylbiguanide hydrochloride and N1,N1,N5,N5-Tetrakis(methyl)-biguanide hydrochloride respectively. The tight binding can be explained in terms of hydrogen bonding with target protein. All the four compounds were involved in the hydrogen bonding with a residue ARG-220 and GLU-249. Metformin hydrochloride interacted with GSK 3β forming H-bonds at active site region involving residues ASP-105, HIS-106 and ILE-109 (Figure 1a - see PDF). The interaction of 1-Carbamimidoyl-1, 2-dimethylguanidine; hydrochloride and GSK 3β involved two hydrogen bonds, with the residue TYR-222 and GLU-249 of GSK 3β (Figure 1b - see PDF). Two-bonds were formed by interaction of N1, N1-Dimethyl-N5-methylbiguanide hydrochloride with GSK 3β involving the residues ARG-220 and TYR-221(Figure 1c - see PDF). The interaction of N1, N1, N5, N5-Tetrakis (methyl)-biguanide hydrochloride and GSK 3β involved two hydrogen bonds, residue ARG-220 and GLU-249 (Figure 1d - see PDF). Analysis of these interaction results confirmed that the selected four analogues showed the efficient binding with diabetic target protein GSK 3β like the standard drug metformin. Metformin mainly interact with the amino acids residues TRY-22 and GLU-249 of GSK 3β. The same way the selected analogues also form interaction with these two residues. So it was confirmed that, these analogues might be a potential lead compounds for experimentally validation for diabetes management.

## Conclusion:

We report the molecular docking analysis of four analogues of metformin [1-Carbamimidoyl-1,2-dimethylguanidine hydrochloride, Metformin hydrochloride, N1,N1-Dimethyl-N5-methylbiguanide hydrochloride, and N1,N1,N5,N5-Tetrakis methyl-biguanide hydrochloride with GSK3 for further consideration in drug discovery for T2DM.
